# The complete chloroplast genome sequence of *Camellia ptilophylla* (Theaceae): a natural caffeine-free tea plant endemic to China

**DOI:** 10.1080/23802359.2018.1457996

**Published:** 2018-04-01

**Authors:** Weixi Li, Fen Xing, Wei Lun Ng, Yubing Zhou, Xianggang Shi

**Affiliations:** State Key Laboratory of Biocontrol and Guangdong Provincial Key Laboratory of Plant Resources, School of Life Sciences, Sun Yat-sen University, Guangzhou, China

**Keywords:** *Camellia ptilophylla*, Theaceae, complete chloroplast genome, endemic to China, caffeine-free

## Abstract

*Camellia ptilophylla* is a natural caffeine-free tea plant endemic to China with high commercial and therapeutic values. Here, we report the complete chloroplast genome assembled using Illumina pair-end sequencing data. The chloroplast genome was 157,097 bp in length, with a large single copy (LSC) region of 86,631 bp, a small single copy (SSC) region of 18,286 bp, separated by two inverted repeat (IR) regions of 26,090 bp each. It contains a total of 132 genes, with an overall GC content of 37.3%. The phylogenetic analysis showed that *C. ptilophylla* is sister to a congeneric species, *C. reticulata*.

Nowadays, as one of most famous beverages in the world, tea is well known for its extraordinary abilities to promote health. However, like coffee, the caffeine in tea can excite nerves, and overdose of tea could lead to some side effects (Mohanpuria et al. 2010). *Camellia ptilophylla* Chang, commonly referred to as the cocoa tea plant, is a wild tea tree endemic to China that contains theobromine instead of caffeine (Chang et al. 1988). Previous studies have also found that cocoa tea has stronger anti-oxidant properties and positive health effects (Peng et al. 2010; Li et al. 2012). The plant is mainly distributed in the Longmen County, Guangdong Province, China (Chang and Ren 1998). In recent years, habitat degradation and loss have resulted in the decrease of population size of the species, further calling for efforts to conserve this valuable wild tea resource. To gain a better insight into its genetics and genomics and thus contribute to its conservation, we assembled and characterized the complete chloroplast genome of *C. ptilophylla* in this study.

Total DNA was isolated from fresh leaves of an individual of *C. ptilophylla*, collected from Nankunshan Nature Reserve, Longmen County, Guangdong Province (23°38′5.21′′ N, 113°52′56′′ E). The voucher specimen (Shi XG 171203) was deposited in the Sun Yat-sen University Herbarium (SYS). Genome sequencing was performed on an Illumina Hiseq X Ten platform generating paired-end reads of 150 bp. In total, 5.99 Gb short sequence data were obtained and used to assemble the chloroplast genome using NOVOPlasty (Dierckxsens et al. 2017). A partial chloroplast *atp*B sequence of the congeneric *C. leptophylla* (GenBank accession number NC_024660) was used as the seed sequence. The genes were annotated using DOGMA (Wyman et al. 2004).

The complete chloroplast genome of *C. ptilophylla* (GenBank accession MG797642) was 157,097 bp in size, containing a pair of inverted repeats (IRs) of 26,090 bp each, separated a large single copy (LSC) region of 86,631 bp and a small single copy (SSC) region of 18,286 bp. The chloroplast genome contained 132 genes, including 90 protein-coding genes, 34 transfer RNA genes, and eight ribosomal RNA genes. Most of the genes occur as single-copy in the LSC or SSC, while 19 gene species had two copies in the IRs. The overall GC content of the chloroplast genome was 37.3%.

Phylogenetic analysis was performed using complete chloroplast genomes from 10 species in the order Ericales including *C. ptilophylla*, and one species from Cornales (*Cornus controversa*) as the outgroup species. The chloroplast genome sequences were aligned using MAFFT (Katoh and Standley 2013). Phylogenetic analysis using the maximum likelihood algorithm was conducted with RAxML (Stamatakis 2014) implemented in Geneious ver. 10.1 (http://www.geneious.com, Kearse et al. 2012). The result showed that *C. ptilophylla* is sister to a congeneric species, *C. reticulata*, with 100% bootstrap support and all species from the family Theaceae were clustered into a monophyletic group (Figure 1).

**Figure 1.**
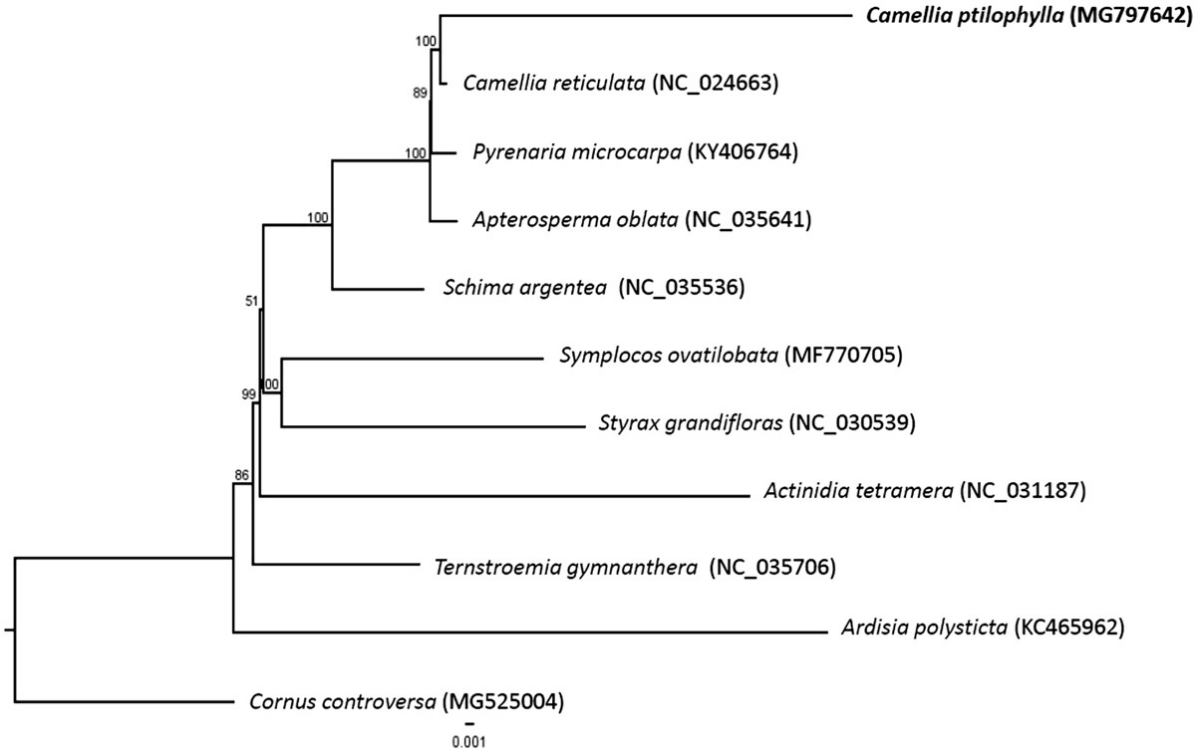
Maximum-likelihood tree based on the sequences of 11 complete chloroplast genomes. Numbers in the nodes are bootstrap support values from 1000 replicates. The position of *Camellia ptilophylla* is shown in bold and GenBank accession numbers are listed behind each species name.
